# Applications and Challenges of Implementing Artificial Intelligence in Medical Education: Integrative Review

**DOI:** 10.2196/13930

**Published:** 2019-06-15

**Authors:** Kai Siang Chan, Nabil Zary

**Affiliations:** 1 Medical Education Scholarship and Research Unit Lee Kong Chian School of Medicine Nanyang Technological University Singapore Singapore; 2 Mohammed Bin Rashid University of Medicine and Health Sciences Dubai United Arab Emirates

**Keywords:** medical education, evaluation of AIED systems, real world applications of AIED systems, artificial intelligence

## Abstract

**Background:**

Since the advent of artificial intelligence (AI) in 1955, the applications of AI have increased over the years within a rapidly changing digital landscape where public expectations are on the rise, fed by social media, industry leaders, and medical practitioners. However, there has been little interest in AI in medical education until the last two decades, with only a recent increase in the number of publications and citations in the field. To our knowledge, thus far, a limited number of articles have discussed or reviewed the current use of AI in medical education.

**Objective:**

This study aims to review the current applications of AI in medical education as well as the challenges of implementing AI in medical education.

**Methods:**

Medline (Ovid), EBSCOhost Education Resources Information Center (ERIC) and Education Source, and Web of Science were searched with explicit inclusion and exclusion criteria. Full text of the selected articles was analyzed using the Extension of Technology Acceptance Model and the Diffusions of Innovations theory. Data were subsequently pooled together and analyzed quantitatively.

**Results:**

A total of 37 articles were identified. Three primary uses of AI in medical education were identified: learning support (n=32), assessment of students’ learning (n=4), and curriculum review (n=1). The main reasons for use of AI are its ability to provide feedback and a guided learning pathway and to decrease costs. Subgroup analysis revealed that medical undergraduates are the primary target audience for AI use. In addition, 34 articles described the challenges of AI implementation in medical education; two main reasons were identified: difficulty in assessing the effectiveness of AI in medical education and technical challenges while developing AI applications.

**Conclusions:**

The primary use of AI in medical education was for learning support mainly due to its ability to provide individualized feedback. Little emphasis was placed on curriculum review and assessment of students’ learning due to the lack of digitalization and sensitive nature of examinations, respectively. Big data manipulation also warrants the need to ensure data integrity. Methodological improvements are required to increase AI adoption by addressing the technical difficulties of creating an AI application and using novel methods to assess the effectiveness of AI. To better integrate AI into the medical profession, measures should be taken to introduce AI into the medical school curriculum for medical professionals to better understand AI algorithms and maximize its use.

## Introduction

Artificial intelligence (AI) has evolved sporadically through the years and most recently gained traction with the advent of deep learning and artificial neural networks. The term AI, created by John McCarthy in 1955 [1], is defined as a machine with intelligent behavior such as perception, reasoning, learning, or communication and the ability to perform human tasks [2]. AI is composed of three main paradigms: symbolic (logic based and knowledge based), statistical (probabilistic methods and machine learning), and subsymbolic (embodied intelligence and search). These paradigms address several problem domains (perception, reasoning, knowledge, planning, and communication). The current applications of AI include its use in automotives, finance and economics, medicine and education [3] including medical education, and Google’s search engine.

The application of AI in medicine remains a hot topic of keen interest for researchers and is under constant development and refinement. One such advancement has machines capable of making a radiological diagnosis at an equal or even higher success rate than highly qualified consultants in that particular specialty. Another well-known example is IBM Watson, which has successfully morphed from its triumph in the game of “Jeopardy!” to the field of medical oncology. Apart from the highly publicized role of AI in radiological diagnosis, other applications include use as an adjunct to the ideal management of cancer or chronic illnesses such as chronic mental disorders [4], particularly regarding the choice of medication with the best response and side effect profiles.

Over the past 25 years, there have been significant developments of AI in education [5], with advances such as “teacher bots,” a teaching assistant tasked to deliver content, provide feedback, and supervise progress [6,7]. This increasingly broader use in the field of education has proven to have the potential to help students receive specialized help and identify knowledge gaps, thereby freeing teachers from menial tasks and allowing them to respond to students more effectively and improve the personalized and adaptive teaching process.

**Figure 1:**
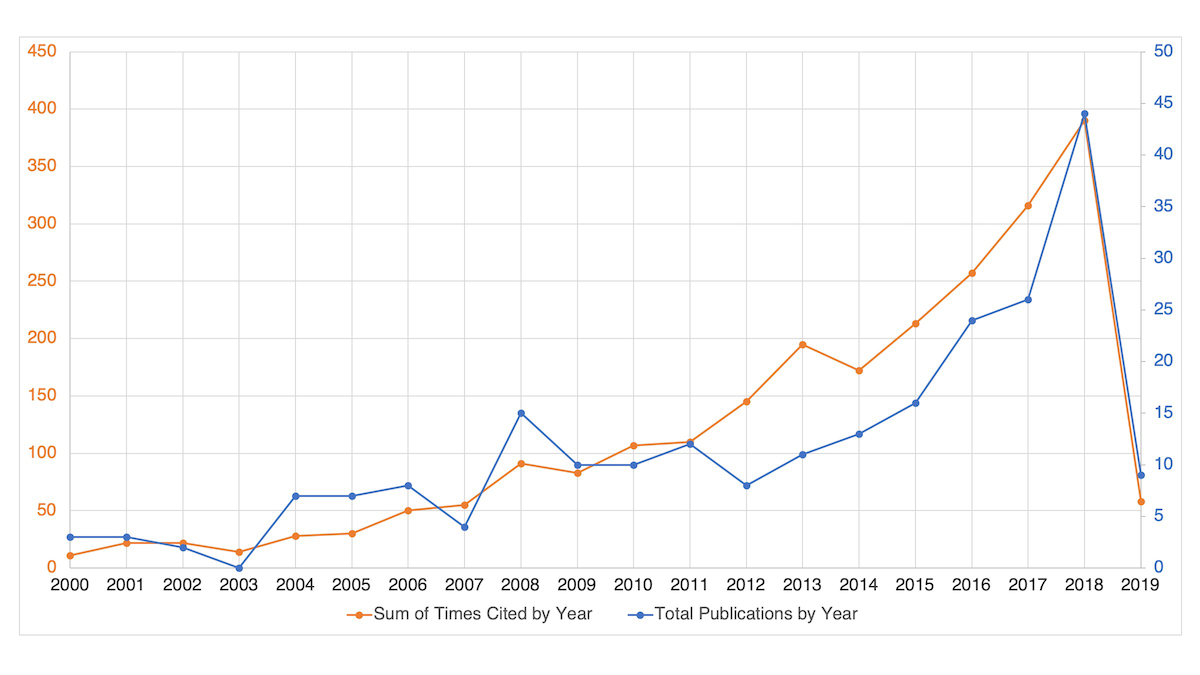
Total publications and sum of times cited by year in the last two decades. Retrieved from Web of Science for artificial intelligence in medical education, dated April 1, 2019.

Medical education encompasses a lifelong learning continuum ranging from undergraduate to postgraduate and specialization training and beyond, also known as “continuing medical education” [8]. It is also applicable to various health care professionals, ranging from doctors to nurses and other allied health care workers. Unlike the field of medicine, there was little interest or advances in AI in medical education during the 1980s, apart from the established projects ATTENDING and GUIDON [9]. Interestingly, a preliminary search in Web of Science for the use of AI in medical education (dated August 14, 2018) demonstrated a growing enthusiasm in this field, with an increase in the number of total publications and times the articles were cited over the last two decades (Figure 1). This reflects an increase in research and development of AI in medical education in recent years.

In this age of rapidly advancing technology, the need to ground novel work on reported research is vital in order to advance the field of AI in medical education. Currently, there are limited articles [9,10] discussing or reviewing the current applications of AI in medical education.

The aim of this study was therefore to review the current reported scholarly work on AI in medical education. Two research questions guided this study:

- How is AI currently used in medical education?
- What are the challenges in implementing AI in medical education?

## Methods

We conducted an integrative review of peer-reviewed literature on AI used in medical education. Integrative reviews are the broadest type of research review method and allow for inclusion of various research designs to more fully understand a phenomenon of interest.

### Data Sources and Search Strategy

We searched Medline (Ovid; 1954 to March 2019), EBSCOhost Education Resources Information Center and Education Source (1983 to March 2019), and Web of Science (1986 to March 2019) to identify articles addressing AI in medical education. We developed the search strategy in collaboration with an academic health sciences librarian. The key search terms were (“artificial intelligence” OR ai OR “machine learning” OR “deep learning”) AND (“medical education” OR “medical student*” OR “medical curriculum” OR “medical school*” OR “medical training”).

### Inclusion and Exclusion Criteria

AI was defined as any technique that enables machines to imitate intelligent human behavior. This includes symbolic (logic based or knowledge based), statistical (probabilistic methods and machine learning), and subsymbolic (embodied intelligence, search, and optimization) AI paradigms covering different problem domains (perception, reasoning, knowledge, planning, and communication). In this study, the chosen medical education focus was on doctors’ professional development across the whole education continuum, from undergraduate, postgraduate, and specialty training to continuing medical education.

Any feature of AI, such as machine learning and deep learning, was included in the search. The exclusion criteria were as follows:

- Articles on other aspects of education apart from medical education
- Articles on use of technology (such as online lectures and computer-based education) without incorporation of AI, or articles with only a brief mention of AI usage
- Article types: reviews, letters, and commentaries
- Full texts of articles available in languages other than English
- Articles published before 1954 due to lack of availability of online archiving of journals

### Selection of Articles for Review

The titles and abstracts of identified articles were screened for the previously identified search criteria, and exclusion criteria were applied. All articles screened to be relevant or inconclusive were assessed in full text. Data such as current application in clinical practice, advantages of such use, and challenges of implementation of AI were extracted from all relevant articles.

### Data Extraction

Data were carefully evaluated and extracted from all the eligible publications. Data retrieved from the studies included the name of the AI application, the study group, the use of AI, and the challenges of implementation as shown in Table 1.

### Analysis of the Articles

Studies relevant to the use of AI in medical education were assessed using the Extension of Technology Acceptance Model and the Diffusion of Innovations theory [48] to explain the adoption of AI in medical education; data were subsequently pooled together and analyzed quantitatively using a statistical software (IBM SPSS Statistics for Windows, version 24.0; Chicago, IL), where relevant.

**Table 1:** Integrative review of the included studies.

Author and year	AI^a^ application	Study group	Use of AI	Challenges of implementation
Clancey and Stanford Univ, 1983 [11]	GUIDON	UG^b^	Guides students to solve problems on infectious diseases using a diagnostic problem-solving approach	The need for a structured set of production rules
Papa et al, 1992 [12]	KBIT^c^	UG	Assess medical students’ diagnostic capabilities	Need to create algorithms for different symptom approach
Eliot and Woolf, 1995 [13]	The Cardiac Tutor	UG	Teaches cardiac resuscitation techniques using a simulation-based tutoring system	Inability to correlate mastery of simulation with the level of ability to perform advanced cardiac life support
Billinghurst et al, 1996 [14]	Prototype sinus surgery	N/A^d^	Provides an intelligent simulation tool or surgical assistant	Requires improved script activation for immediate recognition of surgeon’s actions with an appropriate response
Bourlas et al, 1996 [15]	CARDIO-LOGOS	UG, PG^e^, CME^f^	Assists learners in the recognition and diagnosis of ECG^g^ patterns	Variability in diagnostic criteria of ECG amongst different groups of specialists
Frize and Frasson, 2000 [16]	N/A	N/A	Provides improved learning by detecting the stage of understanding of learners and act as an aid for clinical decision making	N/A
Voss et al, 2000 [17]	LAHYSTOTRAIN	UG, PG, CME	Simulation for training in laparoscopy and hysteroscopy with the provision of feedback	N/A
Stasiu et al, 2001 [18]	CARDIOLOG	UG	Approach to the interpretation of ECG	Narrow knowledge domain restricted to basic cardiac conditions
Kintsch, 2002 [19]	Usage of Latent Semantic Analysis	UG	Assessment of clinical case summaries for medical students	Monetary investments required to develop the algorithm and evaluate the effectiveness of a program
Caudell et al, 2003 [20]	Project TOUCH	N/A	Real-time AI simulation engine in a 3D environment with VR^h^ in a virtual patient	Need to validate the effectiveness of the AI system, network congestions may disrupt group interactions
Crowley and Medvedeva, 2003 [21]	SlideTutor	UG	Teaches diagnostic classification problem solving in dermatopathology	Not suitable for domains where there are no clear prototypical instances or schemas
Michael et al, 2003 [22]	CIRCSIM-Tutor	UG	Develops problem-solving skills on the baroreceptor reflex	Lack of quality explanations for wrong answers
Weidenbach et al, 2004 [23]	EchoComJ	UG, PG, CME	Teaching echocardiography in a simulated environment with feedback provision	Large data input required from real ultrasound images, extremely time-consuming process to develop the algorithm
Kabanza et al, 2006 [24]	TeachMed	UG	Teaches medical students clinical reasoning learning with appropriate feedback/prompts at an individualized pace	Technical difficulties: Having an efficient graph model with minimal loops to improve performance
Suebnukarn and Haddawy, 2006 [25]	COMET	UG	Provides aid in problem-based learning by an appropriate generation of tutorial hints	Inability to assess the effectiveness of COMET unless compared with learning with human tutors, lack of ability to interpret students’ interactions in the chat tool due to lack of natural language processing capabilities
Woo et al, 2006 [26]	CIRCSIM-Tutor	UG	Allows students to practice qualitative causal reasoning in physiology when solving a problem	Inability to interpret and handle expressions of frustration and answers to open questions
Kabassi et al, 2008 [27]	N/A	UG, PG, CME	Develops an adaptive electronic learning system on atheromatosis	The need for capture and analysis of requirements and multidisciplinary input from medical tutors and software engineers
Vicari et al, 2008 [28]	AMPLIA	UG, PG, CME	Supports medical diagnostic reasoning	Students’ lack of confidence in the system's ability to help them to arrive at the correct diagnoses
Kazi et al, 2009 [29]	Extension of COMET	UG	Tutoring system for medical problem-based learning on diabetes, myocardial infarction, and pneumonia	Inferred concepts were mostly overgeneralized or nonrepresentative of the original concepts
Chen and Association for Institutional Research, 2010 [30]	N/A	UG	Construction of a curriculum assessment model using artificial neural network and support vector machine	Trial and error is required to determine training tolerance and configurations for the neural networks
Chieu et al, 2010 [31]	TELEOS project	PG	Teaching the concept of sacroiliac screw fixation in orthopedic surgery	N/A
Lemmon et al, 2011 [32]	N/A	PG	Simulation for junior doctors in the hospital ward setting	The use of an AI chat system based on predefined medical decision-making process, the virtual patient response has limited scalability
Flores et al, 2013 [33]	SimDeCS	UG, PG	Improves diagnostic reasoning in clinical problems in the context of a serious game	Variable reliability due to failure of the AI system
Islam, 2013 [34]	N/A	UG, PG, CME	Analysis of surgical skills in medical students or surgical residents with the provision of feedback	Technical difficulties may limit the effectiveness of the system, eg, the need for high-speed internet connection to upload the video quickly for immediate feedback
Chen et al, 2014 [35]	N/A	UG	Assess students’ notes, identifies their competencies, and aligns them with learning objectives	A large sample size of gold standard annotation by geriatric educators is required
Cao et al, 2015 [36]	CVREA^i^	PG	Provides an effective training platform for anesthetists using a VR environment	The need for a multidisciplinary team: Anesthetists are unable to process data in an engineering way, and engineers are unable to produce clinically interpretable data
Kutafina et al, 2015 [37]	N/A	N/A	Training and evaluation of hand-washing techniques	Randomized controlled trial required to evaluate the effectiveness of the system in comparison with traditional methods of learning
Walkowski et al, 2015 [38]	N/A	UG	Correlation of students’ viewing behaviors of whole-slide images with their test performances	Technical difficulties in development of the machine learning model due to the usage of a different decision trees for each question
Latifi et al, 2016 [39]	N/A	PG	Provide a framework for automated essay scoring using clinical decision-making questions	Need for detailed scoring rubrics and large sample size required for machine learning
McFadden and Crim, 2016 [40]	KBIT	CME	Evaluating the effectiveness of an AI-driven tutor in comparison with didactic lectures	Challenges in assessing the effectiveness of AI due to confounding factors, eg, complete case vignettes provided in the study, which is unlike a real clinical setting
Hamdy et al, 2017 [41]	Virtual Patient Learning	N/A	Provides real patient encounter using an online simulation system to evaluate students’ communication and decision-making abilities	Inability to explore the extent of positive effects on clinical reasoning and communication skills
Khumrin et al, 2017 [42]	N/A	UG	Provides guided learning pathway and personalized feedback for students’ approach to patients presenting with abdominal pain	Difficulty in the provision of effective and individualized feedback for each student
Alonso-Silverio et al, 2018 [43]	N/A	UG, PG	Evaluation of basic laparoscopic skills	Lack of sensitivity to identify trainees who outperform those who are less experienced
Oquendo et al, 2018 [44]	N/A	UG, PG, CME	Performance evaluation of a pediatric laparoscopic suturing task	Difficulty in rating certain scores due to the lack of participation of individuals at the same level of performance
Chen et al, 2018 [45]	Trove radiology resident dashboard	PG	Automates ICD^j^/CPT^k^ classification to provide a more up-to-date dashboard for radiology residents	The rule-based approach had a lack of scalability
Hayasaka et al, 2018 [46]	N/A	N/A	Use of AI-equipped robots as simulated patients	Worry that usage of robots may result in the training of standardized doctors
Kolachalama and Garg, 2018 [47]	N/A	UG	Uses machine learning content in the curriculum to focus on population health and improve patient care	Lack of content specialist in AI to teach students the application of AI knowledge in clinical settings

^a^AI: artificial intelligence.

^b^UG: undergraduate.

^c^KBIT: knowledge-based inference tool.

^d^N/A: not applicable.

^e^PG: postgraduate.

^f^CME: continuing medical education.

^g^ECG: electrocardiogram.

^h^VR: virtual reality.

^i^CVREA: computational VR environment for anesthesia.

^j^ICD: International Statistical Classification of Diseases and Related Health Problems.

^k^CPT: Current Procedural Terminology.

## Results

### Overview

**Figure 2:**
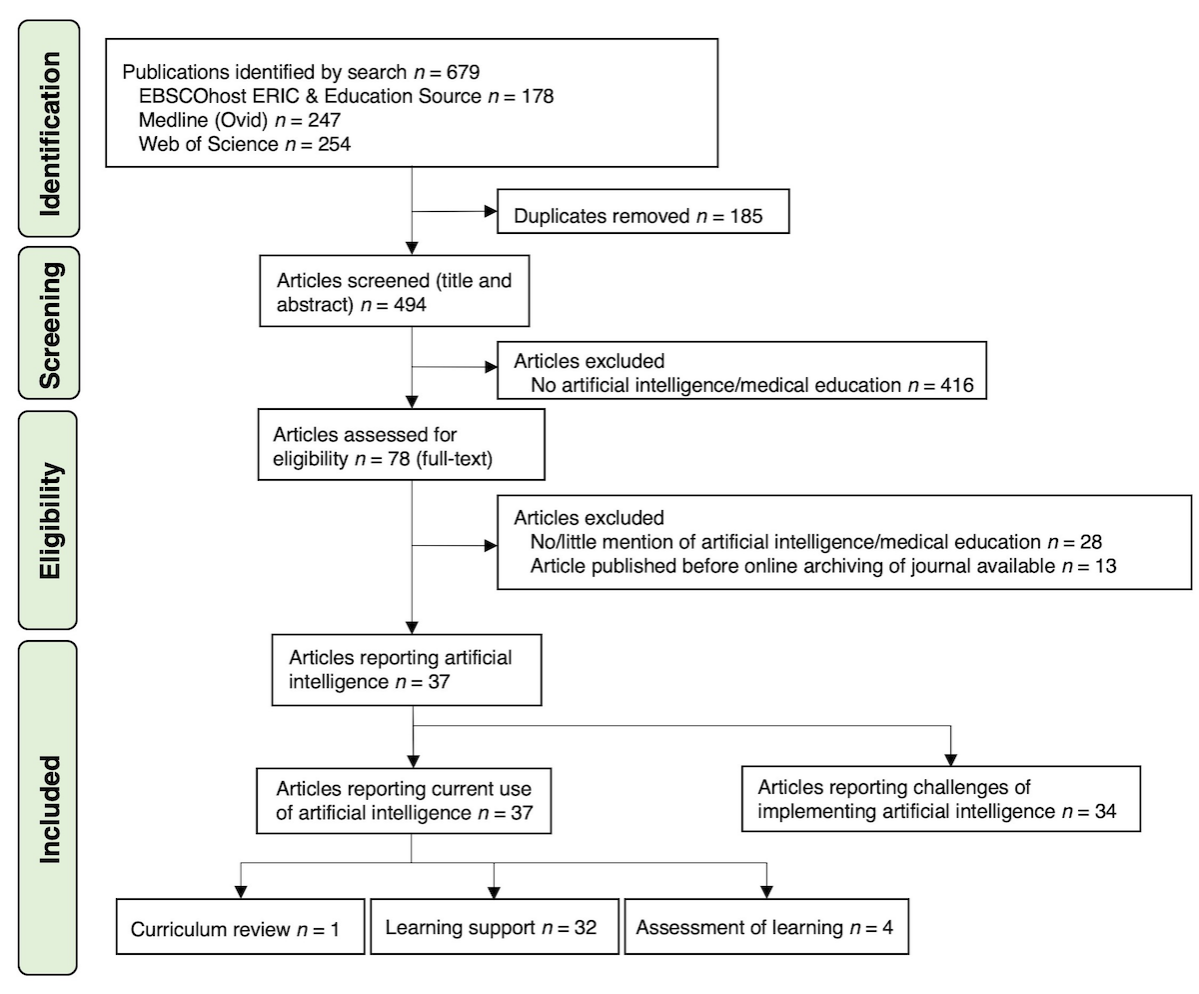
Search strategy for literature on the use of artificial intelligence in medical education in undergraduate, postgraduate, and specialty training in medicine and beyond (continuing medical education). ERIC: Education Resources Information Center.

Our search in the different databases revealed a total of 679 articles (Figure 2). After removal of the duplicates (n=185), the remaining 494 unique articles were screened based on the title and abstract; of those, 416 articles were excluded, as they were not about AI or medical education. Of the remaining 78 articles that were assessed for eligibility, 37 articles were found to be relevant to the use of AI in medical education. Three primary uses of AI in medical education were identified: use of AI as learning support (n=32), assessment of learning (n=4), curriculum review (n=1). Of the 37 articles, 34 articles were found to be relevant to the challenges of implementation of AI in medical education.

### Current Use of Artificial Intelligence in Medical Education

Data gathered from the articles such as advantages of the current uses as well as the challenges of implementation of AI in medical education mentioned in the articles were pooled together and analyzed quantitatively.

One article discussed the use of AI, artificial neural networks (ANN), and support vector machine (SVM) specifically, for assessing the curriculum of medical students. Chen et al [30] described the advantage of ANN and SVM over logistic regression in data analysis: They are more adept models for solving nonlinear problems and establishing relationships between variables. The use of AI in assessing the curriculum of medical education can provide an overview of the effectiveness and students’ satisfaction with the program, which is paramount in training future doctors in medical diagnosis and treatment.

A total of 32 articles discussed the use of AI platforms or systems explicitly designed to improve students’ learning (Multimedia Appendix 1), and 7 articles discussed the use of AI as an adjunct to a virtual environment or simulation for trainees, a majority of which are most relevant to the surgical specialty. The TOUCH project, LAHYSTOTRAIN, and EchoComJ are examples of systems developed using an intelligent tutoring system alongside a virtual reality simulation program. These systems provide added benefits of a virtual environment alongside the benefits of an intelligent tutoring system, including immersive, interactive, and safe environments in a VR simulation, as described by Caudell et al [20].

Four articles examined the use of machine learning models in the assessment of students’ learning (Multimedia Appendix 1). Three articles assessed the use of AI in automated scoring of assignments, and one article [38] assessed the use of machine learning algorithms in predicting the correctness of students’ answers based on their viewing behaviors. Common advantages include an objective assessment of students’ work, more cost-effectiveness and time efficiency, and the ability to provide immediate feedback on their assignment, allowing students to reflect on their work.

**Figure 3:**
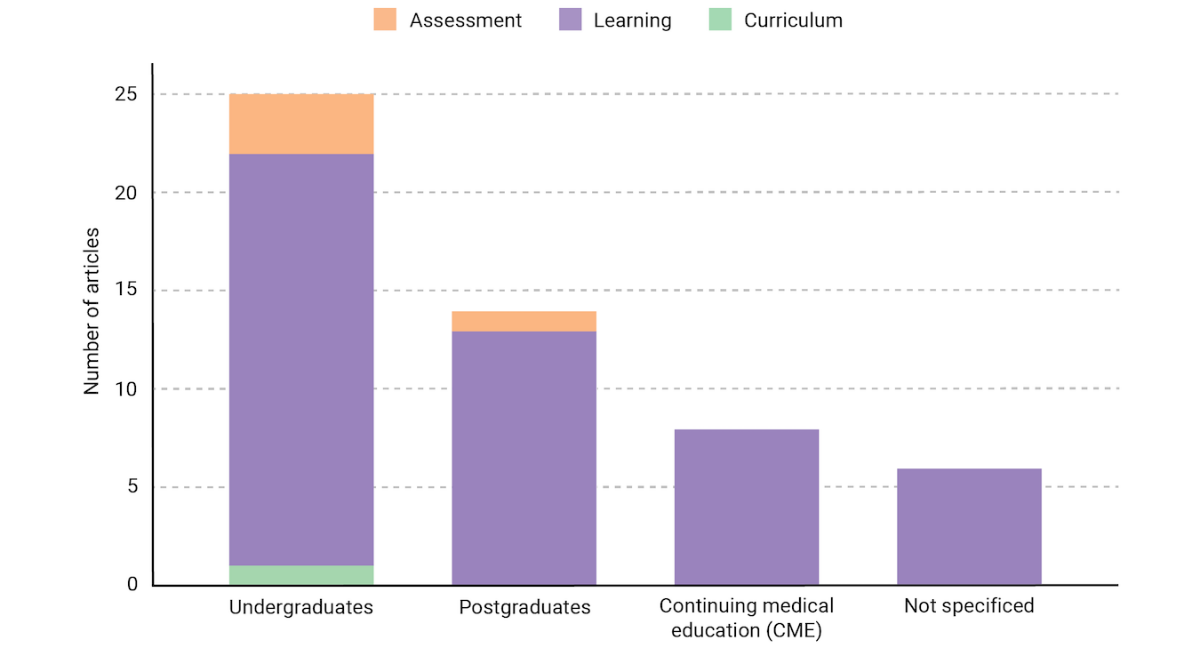
Subgroup analysis showing the number of articles in each focus group for the target audiences.

In addition, there were three main target groups identified in the 37 reviewed articles (Figure 3): medical undergraduates (n=25), postgraduates (n=14), and those continuing medical education (CME; n=8). No specific target group was identified in 6 of the articles; all participants were referred to as “students.”

### Challenges in Implementing Artificial Intelligence in Medical Education

**Figure 4:**
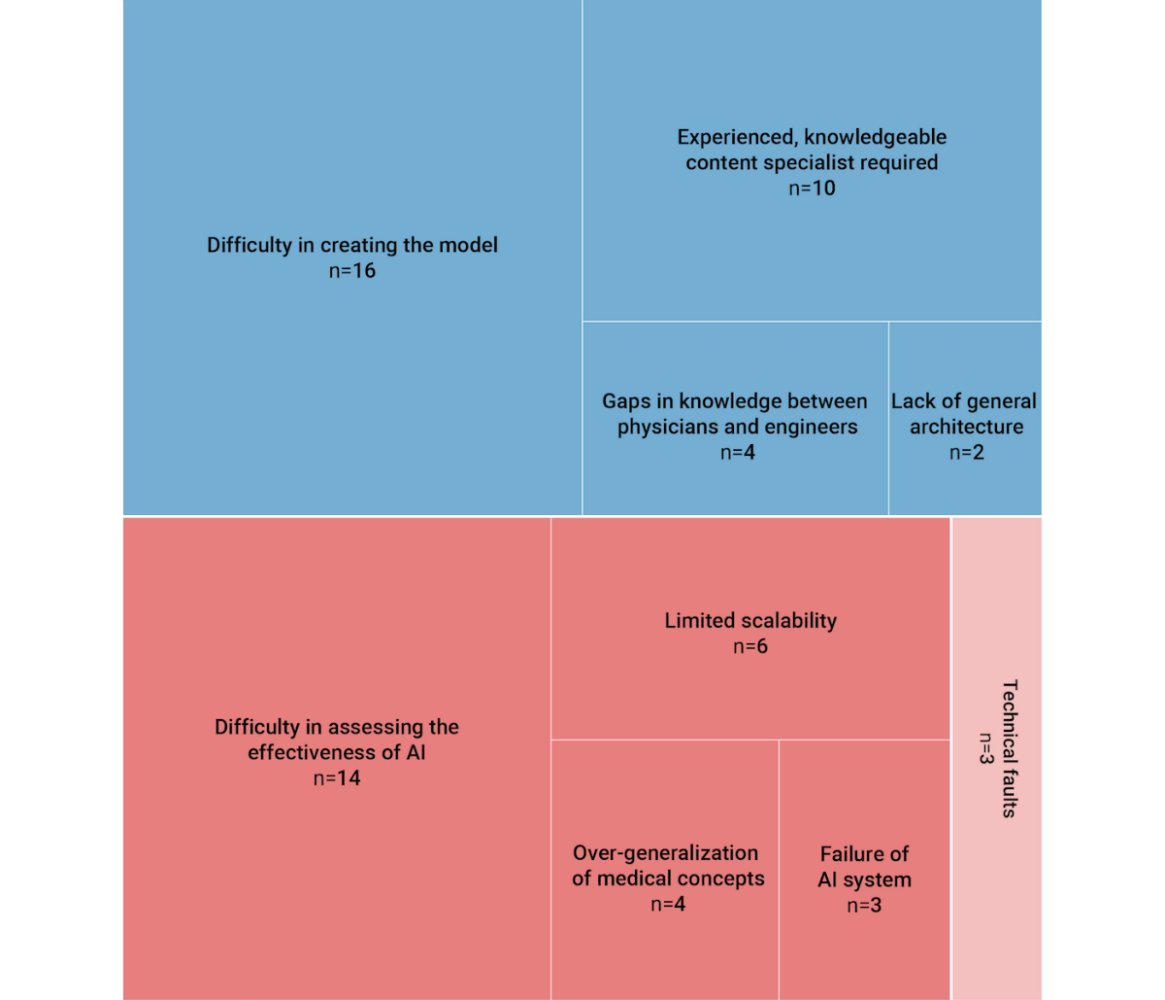
Hierarchical presentation of the challenges of implementation of artificial intelligence (AI) in medical education. The upper blue rectangle shows the proportion of articles in each challenge category in the technical aspects of AI. The lower red rectangle shows the proportion of articles for challenges relating to perceived usefulness (in red) and perceived ease of use (in light red).

One of the elements of Extension of Technology Acceptance Model is perceived usefulness, which includes factors such as difficulty in assessing effectiveness (n=14) and limited scalability (n=6) of the AI system (Figure 4). As discussed by Suebnukarn et al [25], the ideal method of assessing the effectiveness of the system is to conduct a study comparing the use of the AI system with traditional methods of teaching. McFadden et al [40] demonstrated the effectiveness of an AI-driven simulator with a statistically significant improvement in diagnostic accuracy of 22% posttraining as compared to a multimedia-based, expert-led training with a nonstatistical improvement of 8%. Limited scalability of the AI system refers to the narrow range of application of any developed AI system, as the expert models are usually constructed and applicable to a particular specialty of medicine or medical condition.

The critical elements of the Diffusion of Innovations model used to assess the challenges include innovation, communications channel, time, and the social system. The challenges described in the articles mainly centered around the innovation itself (Figure 4). This includes the technological difficulties in acquiring a large sample size needed for the development of the model (n=16), the requirement for a qualified and experienced content specialist to design the curriculum for machine learning (n=10), and the communication challenges relative to the knowledge gap between physicians and engineers (n=4).

In addition, two articles [16,34] discussed the issue of privacy and confidentiality and raised the concern of patient confidentiality when providing data used for an expert system, whereas another study [34] described the measures taken to secure user data.

## Discussion

In this study, we reported the roles and advantages of AI in medical education as well as the challenges that have been hindering the widespread implementation of AI in the medical education community. This section will discuss the main findings.

### Current Use of Artificial Intelligence in Medical Education

A review of the curriculum is an administrative and arduous process, which strongly speaks to the need for machine automation to ease the process. Interestingly, only one of the reviewed articles [30] described the use of AI for medical curriculum review. There is a lack of use of AI in curriculum review despite the advantages AI may have over traditional methods, such as the use of a logistic regression model. Examples of these advantages include the ability of ANNs to solve multidimensional problems, provide greater classification accuracy, and establish strong relationships between variables. One plausible reason for the lack of adoption of AI in curriculum review is the limited digitalization in medical education learning management systems, which is essential for creation of a curriculum map. The digitalization of the curriculum is not possible across all institutions, mainly due to financial constraints [49]. Notably, in Canada and the United Kingdom [50], the majority of medical schools are building curriculum maps. Evidently, as shown in the Results section above (Figure 4), a large pool of data is required to adequately support the development of the model for an AI system. Currently, there are two main approaches to obtaining data—accessing records from prior digitalization of the curriculum and transferring hard copy data into a soft copy, which is a time-consuming process. The latter may well explain the lack of interest in the application of AI with respect to the curriculum in medical education.

The majority of the articles reviewed (32/37) centered around learning and knowledge development. Here, the main reason for AI use (Table 2) was its ability to provide immediate feedback. As highlighted by Hattie et al [51], feedback is critical for identifying learning goals and knowledge gaps. Students need to know how they are performing in order to take measures to improve themselves. However, the provision of feedback is a challenging task in clinical contexts. In a study conducted by Hewson et al [52], 80% of the residents surveyed reported never having or infrequently receiving corrective feedback on their performance. An expert system, on the other hand, can provide immediate and formative feedback on students’ performance.

Interestingly, the question remains: Would that compromise the quality of the feedback received? Useful feedback should essentially assist students in identifying conceptual misunderstandings, critique their performance, and be structured enough to help students achieve their learning objectives [53]. One of the limitations of automated and immediate feedback provision with AI is limitation in the quality of the feedback [18], as the feedback generated is based on the knowledge base and model of the AI system, which, as of now, has room for improvement.

In the subgroup analysis of the articles on learning, medical undergraduates were the primary target audience (21/32) with a lesser focus on CME. This is an exciting finding because undergraduate education forms only a small proportion of a doctor’s professional development. A plausible reason for this finding is the lack of a structured curriculum for CME, with a strong emphasis on professional self-regulation [54]. The development of an AI system requires expert domain knowledge to equip the system with the appropriate curriculum knowledge for contextually driven education.

Without a structured curriculum, it is difficult to select a knowledge base for the AI system, which may, in part, explain the lower prevalence of AI use in CME as compared to that in medical undergraduates. Another possible reason for the preferred focus of AI in undergraduate medical education is that it enables shaping of students’ learning at an earlier point of their medical career. A study by Shin et al [55] demonstrated that undergraduates who adopted problem-based learning are more up to date in medical information as compared to their counterparts who experienced a traditional curriculum. The use of AI enhances problem-based learning, because it provides step-by-step guidance with the appropriate feedback, possibly explaining the preference for targeting medical undergraduates.

Similar to the use of AI in curriculum delivery, only a minority of the articles (4/37) discussed its use in assignments, which begs the question: Why? There are many advantages to using AI in assessments, not the least of which is the ability to provide immediate and formative feedback to students (2/4), which is also the most common reason for the use of AI in learning (21/32). However, the reason for its lack of use is also likely related to the lack of digitalization, explained above in the reasons for the lack of current use of AI for curriculum review. There are many forms of examination in medicine, the most common of which is the written examination and the Objective Structured Clinical Examinations [56]. These methods of assessment are usually conducted offline using pen and paper. 

**Table 2:** Overview of the current uses of artificial intelligence in medical education identified from review of 37 full-text articles.

Focus and advantages of use		Total number of articles
**Curriculum**		
	Comprehensive analysis of the curriculum	1
**Learning**		
	Feedback for learning	21
	Evaluation of the learning process with guided learning pathway	18
	Decreased costs	8
	No harm to patients	6
	Less teacher supervision required	3
**Assessment**		
	Quicker assessment	4
	Objective assessment	3
	Feedback on assessment	2
	Decreased costs	1

Lately, there has been a move to conduct these examinations online. However, there are still several challenges that need to be addressed, including the issue of secured communication and avenues for cheating [57]. Without the digitalization of examinations, it remains an arduous task to transfer hard copy examination results into soft copy to meet the data pool requirements necessary to develop an AI system. In addition, the sensitive nature of summative assessments and examinations limits the use of AI: A malfunction or improper coding of the AI system may cause the results to be incorrect, which may have dire consequences on the students involved. In this regard, AI may be better used in areas in which human performance would increase when assisted by AI and when humans are unable to perform by themselves, such as in adaptive assessment and programmatic assessment. In adaptive assessments, the selection of the questions to follow depends on the user’s answer to the previous questions, such that the difficulty of the questions is tailored to each individual [58]. Programmatic assessment involves the use of an AI system to design an assessment program tailored to optimize learning outcomes and ensure curriculum quality at a systemic level [59]. These are two alternative uses of AI that may be considered part of augmenting assessment in medical education. This is in addition to AI’s role in the marking essays, as described in the reviewed articles.

### Challenges of Implementation of Artificial Intelligence in Medical Education

This section will discuss the two main groups of challenges hindering the implementation of AI: (1) limitations in the perceived usefulness of AI and (2) the technical difficulties with the development of AI applications.

Difficulty in assessing the effectiveness of the AI application was the most commonly reported challenge (14/34). To prove the effectiveness of the AI system, an ideal approach would involve scientific rigor and the ability to explore what AI does (“explainability”). The issue of “explainability” is specific for deep learning, which is a subset of AI. Due to the nonlinear nature of deep learning, there is often no explanation of how the AI system arrives at the answer or prediction [60]. However, an explanation of the thought process is crucial for students’ learning, especially in medical education, where clinical reasoning forms the foundation of a doctor’s professional development.

What is clinical reasoning? It was initially described by Barrows [61] as “the cognitive process that is necessary to evaluate and manage a patient's medical problem.” Clinical problems are often ill-structured and multifaceted, which explains the need for a comprehensive history from a patient. These factors are then taken into consideration, and a clinical impression is formed, which then enables the construction of differential diagnoses. As this is a much more complex process than providing a simple diagnosis label based on the symptom complex of a patient, the use of deep learning reaches the cognitive limits to aid in medical education. In addition, to objectively prove the effectiveness of AI, studies need to compare the use of AI with traditional methods of teaching. These studies require a large sample size for the results to be probabilistic. Clear surrogate markers such as pretest and posttest scores are fundamental to analyze the results objectively. Study subjects should also have a similar level of understanding of the topic taught before any intervention. As a result, limited studies [40] have been conducted to assess the effectiveness of AI in medical education.

An essential aspect of developing an AI system is the need for a multidisciplinary team (included in 4 of 34 studies; Figure 4), including educational experts, data scientists for management of the large pool of data, physicians for ensuring the clinical relevance, and accuracy of the AI system. Engineers and data scientists are more focused on the accuracy of the AI system to determine how likely the system is to predict a result correctly. However, this still may have little clinical and educational relevance. If this relevance is to be attained, the medical domain and educational experts will increasingly need to work in concert with data scientists in order to develop AI systems that are both accurate and effective in medical education.

### The Learners’ Data Integrity

Worth noting is that only two of the reviewed articles [16,34] raised the issue of privacy and confidentiality. In the world of digitalization, data protection is paramount. This is seen in the rise of statutory laws such as the Data Protection Act 2018 (United Kingdom) [62] and the Personal Data Protection Act 2012 (Singapore) [63]. Data are protected, especially if AI practice is conducted in commercial settings where companies profit from gathering data. Concurrently, there is a need to develop novel models that allow access to educational data for the development of AI applications [64].

For example, data of learners undergoing CME may be used as a factor for determining performance. Any leakage or manipulation of these data may adversely affect the promotion of doctors. The lack of a robust data protection measure places learners’ data (which is often used to train AI algorithms) at risk and may well lead to societal rejection of the use of AI in medical education, a key element in the Diffusion of Innovations theory. It is therefore necessary to consider data security in addition to the perceived usefulness and technical difficulties of an AI system.

### The Ability of Artificial Intelligence Systems to Address Ethical Issues

Medical education emphasizes on the importance of ethical judgment. Students need to be taught about how to approach ethical issues as well as the need to have informed discussions regarding the approach to ethical issues and decision making. In this era of increasingly complex health care and patient-centric care, clinical decisions should not be made solely on technical and medical grounds [65]. Other factors ranging from patients’ expectations and values to resource allocation and medical futility also need to be addressed. However, ethical decisions are often difficult to make even for highly trained and experienced doctors. This calls for the development of clinical ethics committees [66] that aim at addressing ethical issues that arise within patient care scenarios. In the context of ethical issues, which are multifactorial and highly situational, the use of AI in medical education can be limited in some contexts. For example, an intrinsic limitation underlying the use of AI is the inability to show concern [67]. Wartman and Combs emphasize on the importance of empathy among physicians toward their patients. If AI targets medical students early in their medical education, there is a commensurate need to balance the teaching and learning they receive from both health professionals and AI systems in order to ensure these students experience an appropriate and balanced exposure to the “art of medicine.”

### Introduction of Artificial Intelligence Into the Medical Profession

One of the difficulties experienced in the implementation of AI in medical education is the gap in knowledge between physicians and engineers (Figure 4), which leads to this question: Should AI be introduced to medical professionals and trainees, and if so, how? This is the ideal situation that would solve one of the significant difficulties of implementing AI. This has been recently discussed by Kolachalama and Garg [47]; the current medical school curriculum is unable to accommodate AI due to two main reasons—insufficient time and lack of expertise. Although we acknowledge the difficulty of teaching AI in the short 5-6 years of medical school, tweaks can be made to the curricula to introduce the concepts of AI alongside traditional medical school teaching. For instance, AI applications such as CARDIO-LOGOS (Table 1) can be introduced to teach clinical students the diagnostic approach of reading electrocardiograms, but students can also be simultaneously taught the algorithm the machine uses to maximize their learning when introduced to the application. Other techniques to introduce AI include abstinence from jargon and highlighting the application of AI in the diagnosis and management of real patients [47]. Another factor that has been raised earlier is the lack of expertise to teach AI in the medical profession. An easy way to do so is to collaborate with engineering and computing faculties and seek their professional opinions. Interfaculty collaborations and competitions can also be held in universities to promote interaction between students and peers and allow the sharing of expertise across different fields such as health care hackathons, which have been increasing in recent years [68].

### Future Research

Based on the findings from our review, we propose that future research should focus on assessing the effectiveness of AI in medical education. Only one study that reviewed expert-led training in rheumatology has thus far shown the benefit of the use of an AI-driven system as compared to traditional methods. Given that the diagnostic approach varies across specialties, intensive and time-consuming research is still needed in every subspecialty to truly determine the success of AI systems as compared to traditional approaches.

With the increased use of AI systems made possible through the evolving digitalization of the medical curriculum and collaboration between data scientists and physicians, the issue of data protection will need to be researched with an emphasis on how best to improve data security and increase users’ confidence of the use of AI applications.

As technology continues to advance, the potential uses of AI will continue to increase in medical education. One such development will be the use of AI, combined with immersive technologies such as virtual reality and augmented reality. As presented in our results, such studies have already been reported. Further research should explore more complex adaptations of AI in medical education.

### Limitations

The scope of this review covers a broad spectrum of the current applications of AI in medical education. In the field of medicine, where the practices of each subspecialty vary tremendously, the use of AI in education may also vary. It may therefore be too early to make an overarching statement about the benefits of AI in medical education. One limitation in the interpretation of the results is the high proportion of articles on the use of AI as a support for learning, as compared to its use in support of the development and review of the curriculum. Another is in the summative assessment of learners’ performance. Although these may be representative of the current uses of AI, the conclusions drawn from the uses of AI in the curriculum and assessment may be inconsequential due to the low number of studies reviewed.

### Conclusions

This review identified the current uses of AI in medical education, which include curriculum assessment and improvement of students’ learning, with research mainly existing on the latter. The studies also highlighted the main challenges hindering the implementation of AI in medical education, which relate to how best to assess the effectiveness of AI and to manage the technical difficulties associated with the effective and productive development of an AI system.

## Appendix

Multimedia Appendix 1 Data used to generate Figures 3,4 and list of articles for each aspect presented.

## Abbreviations

AI: artificial intelligence
ANN: artificial neural networks
CME: continuing medical education
CPT: Current Procedural Terminology
CVREA: computational VR environment for anesthesia
ECG: electrocardiogram
ICD: International Statistical Classification of Diseases and Related Health Problems
KBIT: knowledge-based inference tool
PG: postgraduate
SVM: support vector machine
UG: undergraduate
VR: virtual reality

## References

[1] McCarthy J, Minsky ML, Rochester N, Shannon CE (2006). AI magazine.

[2] Nilsson NJ (1998). Artificial Intelligence: A New Synthesis.

[3] Russell S, Norvig P (2018). Artificial intelligence: a modern approach, global edition.

[4] Hamet P, Tremblay J (2017). Artificial intelligence in medicine. Metabolism.

[5] Roll I, Wylie R (2016). Evolution and revolution in artificial intelligence in education. International Journal of Artificial Intelligence in Education.

[6] Bayne S (2015). Teacherbot: interventions in automated teaching. Teaching in Higher Education.

[7] Botrel L, Holz E, Kübler A (2015). Brain Painting V2: evaluation of P300-based brain-computer interface for creative expression by an end-user following the user-centered design. Brain-Computer Interfaces.

[8] Bashook P, Parboosingh J (1998). Recertification and the maintenance of competence. BMJ.

[9] Lillehaug S, Lajoie S (1998). AI in medical education--another grand challenge for medical informatics. Artif Intell Med.

[10] Wartman S, Combs C (2018). Medical Education Must Move From the Information Age to the Age of Artificial Intelligence. Acad Med.

[11] Clancey W (1983). Stanford Univ CADoCS. GUIDON. Technical Report.

[12] Papa F, Shores J, Texas Coll of Osteopathic Medicine FW (1992). Expert Systems Based Clinical Assessment and Tutorial Project. Reports.

[13] Eliot C, Woolf B (1995). An adaptive Student Centered Curriculum for an intelligent training system. User Model User-Adap Inter.

[14] Billinghurst M, Savage J, Oppenheimer P, Edmond C (1996). The expert surgical assistant. An intelligent virtual environment with multimodal input. Stud Health Technol Inform.

[15] Bourlas P, Giakoumakis E, Koutsouris D, Papakonstantinou G, Tsanakas P (1996). The CARDIO-LOGOS system for ECG training and diagnosis. Technol Health Care.

[16] Frize M, Frasson C (2000). Decision-support and intelligent tutoring systems in medical education. Clin Invest Med.

[17] Voss G, Bockholt U, Los Arcos J L, Müller W, Oppelt P, Stähler J (2000). LAHYSTOTRAIN intelligent training system for laparoscopy and hysteroscopy. Stud Health Technol Inform.

[18] Stasiu RK, De Britto J, Da Silva Dias J, Scalabrin E (2001). Teaching of electrocardiogram interpretation guided by a tutorial expert. Proceedings 14th IEEE Symposium on Computer-Based Medical Systems.

[19] Kintsch W (2002). The potential of latent semantic analysis for machine grading of clinical case summaries. J Biomed Inform.

[20] Caudell T, Summers K, Holten J, Hakamata T, Mowafi M, Jacobs J, Lozanoff Beth K, Lozanoff Scott, Wilks David, Keep Marcus F, Saiki Stanley, Alverson Dale (2003). Virtual patient simulator for distributed collaborative medical education. Anat Rec B New Anat.

[21] Crowley R, Medvedeva O (2003). A general architecture for intelligent tutoring of diagnostic classification problem solving. AMIA Annual Symposium Proceedings.

[22] Michael J, Rovick A, Glass M, Zhou Y, Evens M (2010). Learning from a Computer Tutor with Natural Language Capabilities. Interactive Learning Environments.

[23] Weidenbach M, Trochim S, Kreutter S, Richter C, Berlage T, Grunst G (2004). Intelligent training system integrated in an echocardiography simulator. Comput Biol Med.

[24] Kabanza F, Bisson G, Charneau A, Jang T (2006). Implementing tutoring strategies into a patient simulator for clinical reasoning learning. Artif Intell Med.

[25] Suebnukarn S, Haddawy P (2006). A Bayesian approach to generating tutorial hints in a collaborative medical problem-based learning system. Artif Intell Med.

[26] Woo C, Evens M, Freedman R, Glass M, Shim L, Zhang Y, Zhou Yujian, Michael Joel (2006). An intelligent tutoring system that generates a natural language dialogue using dynamic multi-level planning. Artif Intell Med.

[27] Kabassi K, Virvou M, Tsihrintzis G, Vlachos Y, Perrea D (2008). Specifying the personalization reasoning mechanism for an intelligent medical e-learning system on Atheromatosis: An empirical study. IDT.

[28] Vicari R, Flores C, Seixas L, Gluz J, Coelho H (2008). AMPLIA: A Probabilistic Learning Environment. International Journal of Artificial Intelligence in Education.

[29] Kazi H, Haddawy P, Suebnukarn S (2009). Expanding the Space of Plausible Solutions in a Medical Tutoring System for Problem-Based Learning. International Journal of Artificial Intelligence in Education.

[30] Chen CK (2010). Curriculum Assessment Using Artificial Neural Network and Support Vector Machine Modeling Approaches: A Case Study. IR Applications.

[31] Chieu VM, Luengo V, Vadcard L, Tonetti J (2010). Student Modeling in Orthopedic Surgery Training: Exploiting Symbiosis between Temporal Bayesian Networks and Fine-Grained Didactic Analysis. International Journal of Artificial Intelligence in Education.

[32] Lemmon C, Siu MLC, Vincent HV, Hamilton J (2011). The Importance of Humans in Simulation: Allowing the Lure of Technology to Drive Development. Proceedings of the European Conference on Games Based Learning.

[33] Flores C, Barros P, Cazella S, Bez M (2013). Leveraging the Learning Process in Health through Clinical Cases Simulator. IEEE Xplore.

[34] Islam G (2013). Informatics Approach to Improving Surgical Skills Training.

[35] Chen Y, Wrenn J, Xu H, Spickard A, Habermann R, Powers J (2014). Automated Assessment of Medical Students' Clinical Exposures according to AAMC Geriatric Competencies. AMIA Annual Symposium Proceedings.

[36] Cao X, Zhang P, He J, Huang G (2015). Building Computational Virtual Reality Environment for Anesthesia.

[37] Kutafina E, Laukamp D, Jonas S (2015). Wearable Sensors in Medical Education: Supporting Hand Hygiene Training with a Forearm EMG. Stud Health Technol Inform.

[38] Walkowski S, Lundin M, Szymas J, Lundin J (2015). Exploring viewing behavior data from whole slide images to predict correctness of students' answers during practical exams in oral pathology. J Pathol Inform.

[39] Latifi S, Gierl M, Boulais A, De Champlain André F (2016). Using Automated Scoring to Evaluate Written Responses in English and French on a High-Stakes Clinical Competency Examination. Eval Health Prof.

[40] McFadden P, Crim A (2016). Comparison of the Effectiveness of Interactive Didactic Lecture Versus Online Simulation-Based CME Programs Directed at Improving the Diagnostic Capabilities of Primary Care Practitioners. J Contin Educ Health Prof.

[41] Hamdy H, Al-Moslih A, Tavarnesi G, Laus A (2017). Virtual patients in problem-based learning. Med Educ.

[42] Khumrin P, Ryan A, Judd T, Verspoor K (2017). Diagnostic Machine Learning Models for Acute Abdominal Pain: Towards an e-Learning Tool for Medical Students. Stud Health Technol Inform.

[43] Alonso-Silverio G, Pérez-Escamirosa Fernando, Bruno-Sanchez R, Ortiz-Simon J, Muñoz-Guerrero Roberto, Minor-Martinez A, Alarcón-Paredes Antonio (2018). Development of a Laparoscopic Box Trainer Based on Open Source Hardware and Artificial Intelligence for Objective Assessment of Surgical Psychomotor Skills. Surg Innov.

[44] Oquendo Y, Riddle E, Hiller D, Blinman T, Kuchenbecker K (2018). Automatically rating trainee skill at a pediatric laparoscopic suturing task. Surg Endosc.

[45] Chen H, Gangaram V, Shih G (2019). Developing a More Responsive Radiology Resident Dashboard. J Digit Imaging.

[46] Hayasaka Y, Fujikura T, Kashimura M (2018). Expectations for the Next Generation of Simulated Patients Born from Thoughtful Anticipation of Artificial Intelligence-Equipped Robot. J Nippon Med Sch.

[47] Kolachalama V, Garg P (2018). Machine learning and medical education. npj Digital Med.

[48] Taherdoost H (2018). A review of technology acceptance and adoption models and theories. Procedia Manufacturing.

[49] Frehywot S, Vovides Y, Talib Z, Mikhail N, Ross H, Wohltjen H, Bedada Selam, Korhumel Kristine, Koumare Abdel Karim, Scott James (2013). E-learning in medical education in resource constrained low- and middle-income countries. Hum Resour Health.

[50] Willett T (2008). Current status of curriculum mapping in Canada and the UK. Med Educ.

[51] Hattie J, Timperley H (2016). The Power of Feedback. Review of Educational Research.

[52] Hewson M, Little M (1998). Giving feedback in medical education: verification of recommended techniques. J Gen Intern Med.

[53] Mason BJ, Bruning RH (2001). Providing feedback in computer-based instruction: What the research tells us. CLASS Research Report.

[54] Peck C, McCall M, McLaren B, Rotem T (2000). Continuing medical education and continuing professional development: international comparisons. BMJ.

[55] Shin J, Haynes R, Johnston M (1993). Effect of problem-based, self-directed undergraduate education on life-long learning. CMAJ.

[56] Epstein R (2007). Assessment in medical education. N Engl J Med.

[57] Sarrayrih M, Ilyas M (2013). Challenges of online exam, performances and problems for online university exam. International Journal of Computer Science Issues.

[58] Ward J, Gordon J, Field M, Lehmann H (2001). Communication and information technology in medical education. Lancet.

[59] Van Der Vleuten CPM, Schuwirth LWT, Driessen EW, Govaerts MJB, Heeneman S (2015). Twelve Tips for programmatic assessment. Med Teach.

[60] Samek W, Wiegand T, Müller K-R (2017). arXiv Preprint.

[61] Barrows H, Tamblyn R (1980). Problem-based learning: An approach to medical education.

[62] Legislation.gov.uk.

[63] PDPC.

[64] Ellaway R, Topps D, Pusic M (2019). Data, Big and Small: Emerging Challenges to Medical Education Scholarship. Acad Med.

[65] Lo B (2012). Resolving ethical dilemmas: a guide for clinicians.

[66] McLean S (2007). What and who are clinical ethics committees for?. J Med Ethics.

[67] Wartman S, Combs C (2019). Reimagining Medical Education in the Age of AI. AMA J Ethics.

[68] Wang J, Roy S, Barry M, Chang R, Bhatt A (2018). Institutionalizing healthcare hackathons to promote diversity in collaboration in medicine. BMC Med Educ.

