# Supplementary material for: Applications and Challenges of Implementing Artificial Intelligence in Medical Education: Integrative Review
Source: JMIR Med Educ. 2019 Jun 15;5(1):e13930. doi: 10.2196/13930 (PMC6598417; doi:10.2196/13930)
Supplement: Multimedia Appendix 1 [file mededu_v5i1e13930_app1.pdf]

# Applications and Challenges of Implementing Artificial Intelligence in Medical Education: An Integrative Review

## Appendix I

**Overview of current use of AI in medical education; Review of the 37 identified full-text articles revealed three main uses: curriculum, learning and assessment**

| Focus | Advantages of use | Articles | Total number of articles |
|---|---|---|---|
| Curriculum | Comprehensive analysis of the curriculum | [30] | 1 |
| Learning | Feedback for learning | [43], [14], [31], [21], [13], [33], [16], [41], [34], [24], [29], [42], [40], [22], [44], [18], [25], [28], [17], [23], [26] | 21 |
| | Evaluation of the learning process with guided learning pathway | [14], [15], [31], [11], [21], [13], [33], [41], [24], [27], [37], [40], [22], [25], [28], [17], [23], [26], [32], [12] | 18 |
| | Decreased costs | [43], [14], [13], [34], [29], [44], [25], [17] | 8 |
| | No harm to patients | [43], [14], [36], [33], [34], [44] | 6 |
| | Less teacher supervision required | [20], [44], [46] | 3 |
| Assessment | Quicker | [35], [19], [39], [38] | 4 |
| | Objective assessment | [35], [19], [39] | 3 |
| | Feedback on assessment | [19], [39] | 2 |
| | Decreased costs | [39] | 1 |

**Subgroup analysis showing the target audiences of the 37 reviewed articles**

| Focus | Target group | | | |
|---|---|---|---|---|
| | Undergraduates | Post-graduates | Continuing medical education (CME) | Not specified |
| Curriculum | 1 | 0 | 0 | 0 |
| Learning | 21 | 13 | 8 | 6 |
| Assessment | 3 | 1 | 0 | 0 |
| **Total** | **25** | **14** | **8** | **6** |

In addition, there were three main target groups identified in the 37 reviewed articles (Table 3); medical undergraduates (n= 25), postgraduates (n= 14), and Continuing Medical Education (CME) (n= 8). No specific target group was identified in 6 of the articles; they were referred to as "students."

**Overview of challenges of implementation of AI in medical education; Review of the 34 identified full-text articles using the ETAM and DOI theory**

| | Challenges | Articles (No as indicated in the reference list) | Total number of articles |
|---|---|---|---|
| Acceptance of AI as technology | | | |
| Perceived usefulness | Difficulty in assessing the effectiveness of AI | [15], [20], [13], [33], [41], [42], [19], [37], [40], [22], [44], [18], [25], [28] | 14 |
| | Limited scalability | [32], [12], [18], [25], [26], [45] | 6 |
| | Failure of AI system (e.g., suggestions for learning, incorrect information) | [13], [33], [24] | 3 |
| | Over-generalization of medical concepts | [15], [21], [29], [46] | 4 |
| Perceived ease of use | Technical faults, e.g., network errors | [20], [13], [34] | 3 |
| Diffusion of AI as an innovation | | | |
| AI technical aspects | Difficulty in creating the model – large sample size, many trials required | [43], [14], [30], [35], [34], [24], [27], [29], [42], [19], [39], [32], [44], [12], [23], [45] | 16 |
| | Experienced, knowledgeable content specialist required | [14], [34], [19], [44], [18], [25], [38], [23], [26], [47] | 10 |
| | Gaps in knowledge between physicians and engineers | [36], [27], [32], [25] | 4 |
| | Lack of general architecture | [21], [27] | 2 |

# References


1. McCarthy J, Minsky ML, Rochester N, Shannon CE. A proposal for the dartmouth summer research project on artificial intelligence, august 31, 1955. AI magazine. 2006;27(4):12.
2. Nilsson NJ. Artificial intelligence: a new synthesis: Morgan Kaufmann; 1998.
3. Russell SJ, Norvig P. Artificial intelligence: a modern approach: Malaysia; Pearson Education Limited; 2016.
4. Hamet P, Tremblay J. Artificial intelligence in medicine. Metabolism. 2017;69:S36-S40.
5. Roll I, Wylie R. Evolution and revolution in artificial intelligence in education. International Journal of Artificial Intelligence in Education. 2016;26(2):582-99.
6. Bayne S. Teacherbot: interventions in automated teaching. Teaching in Higher Education. 2015;20(4):455-67.
7. Botrel L, Holz E, Kübler A. Brain Painting V2: evaluation of P300-based brain-computer interface for creative expression by an end-user following the user-centered design. Brain-Computer Interfaces. 2015;2(2-3):135-49.
8. Bashook P. Clinical competence and continuing medical education: Lifelong learning to maintain competence. Learning in medicine. 1993:21-41.
9. Lillehaug S-I, Lajoie SP. AI in medical education—another grand challenge for medical informatics. Artificial Intelligence in Medicine. 1998;12(3):197-225.
10. Wartman SA, Combs CD. Medical Education Must Move From the Information Age to the Age of Artificial Intelligence. Academic Medicine. 2018;93(8):1107-9.

11. Clancey WJ, Stanford Univ CADoCS. GUIDON. Technical Report #9. 1983.
12. Papa F, Shores J, Texas Coll. of Osteopathic Medicine FW. Expert Systems Based Clinical Assessment and Tutorial Project. 1992.
13. Eliot C, Woolf BP. An Adaptive Student Centered Curriculum for an Intelligent Training System. User Modeling and User-Adapted Interaction. 1995;5(1):67-86.
14. Billinghurst M, Savage J, Oppenheimer P, Edmond C. The expert surgical assistant. An intelligent virtual environment with multimodal input. Studies in Health Technology & Informatics. 1996;29:590-607.
15. Bourlas P, Giakoumakis E, Koutsouris D, Papakonstantinou G, Tsanakas P. The CARDIO-LOGOS system for ECG training and diagnosis. Technology & Health Care. 1996;3(4):279-85.
16. Frize M, Frasson C. Decision-support and intelligent tutoring systems in medical education. Clinical & Investigative Medicine - Medecine Clinique et Experimentale. 2000;23(4):266-9.
17. Voss G, Bockholt U, Los Arcos JL, Muller W, Oppelt P, Stahler J. LAHYSTOTRAIN intelligent training system for laparoscopy and hysteroscopy. Studies in Health Technology & Informatics. 2000;70:359-64.
18. Stasiu RK, de Britto J, Dias JD, Scalabrin E, Ieee Computer S. Teaching of electrocardiogram interpretation guided by a tutorial expert. Fourteenth Ieee Symposium on Computer-Based Medical Systems, Proceedings. Computer-Based Medical Systems : Proceedings of the Annual Ieee Symposium2001. p. 487-92.
19. Kintsch W. The potential of latent semantic analysis for machine grading of clinical case summaries. Journal of Biomedical Informatics. 2002;35(1):3-7.
20. Caudell TP, Summers KL, Holten Jt, Hakamata T, Mowafi M, Jacobs J, et al. Virtual patient simulator for distributed collaborative medical education. Anatomical Record New Anatomist. 2003;270(1):23-9.
21. Crowley RS, Medvedeva O. A general architecture for intelligent tutoring of diagnostic classification problem solving. AMIA 2003;Annual Symposium Proceedings/AMIA Symposium.:185-9.
22. Michael J, Rovick A, Glass M, Zhou Y, Evens Meie. Learning from a Computer Tutor with Natural Language Capabilities. Interactive Learning Environments. 2003;11(3):233-62.
23. Weidenbach M, Trochim S, Kreutter S, Richter C, Berlage T, Grunst G. Intelligent training system integrated in an echocardiography simulator. Computers in Biology & Medicine. 2004;34(5):407-25.
24. Kabanza F, Bisson G, Charneau A, Jang TS. Implementing tutoring strategies into a patient simulator for clinical reasoning learning. Artificial Intelligence in Medicine. 2006;38(1):79-96.
25. Suebnukarn S, Haddawy P. A Bayesian approach to generating tutorial hints in a collaborative medical problem-based learning system. Artificial Intelligence in Medicine. 2006;38(1):5-24.
26. Woo CW, Evens MW, Freedman R, Glass M, Shim LS, Zhang Y, et al. An intelligent tutoring system that generates a natural language dialogue using dynamic multi-level planning. Artificial Intelligence in Medicine. 2006;38(1):25-46.
27. Kabassi K, Virvou M, Tsihrintzis GA, Vlachos Y, Perrea D. Specifying the personalization reasoning mechanism for an intelligent medical e-learning system on Atheromatosis: An empirical study. Intelligent Decision Technologies-Netherlands. 2008;2(3):179-90.
28. Vicari Rriub, Flores CDdfeb, Seixas Llsub, Gluz JCjub, Coelho Hhdfup. AMPLIA: A Probabilistic Learning Environment. International Journal of Artificial Intelligence in Education (IOS Press). 2008;18(4):347-73.
29. Kazi H, Haddawy P, Suebnukarn S. Expanding the Space of Plausible Solutions in a Medical Tutoring System for Problem-Based Learning. International Journal of Artificial Intelligence in Education. 2009;19(3):309-34.
30. Chen C-K, Association for Institutional R. Curriculum Assessment Using Artificial Neural Network and Support Vector Machine Modeling Approaches: A Case Study. IR Applications. Volume 29. Association for Institutional Research; 2010.
31. Chieu VM, Luengo V, Vadcard L, Tonetti J. Student Modeling in Orthopedic Surgery Training: Exploiting Symbiosis between Temporal Bayesian Networks and Fine-Grained Didactic Analysis. International Journal of Artificial Intelligence in Education. 2010;20(3):269-301.
32. Lemmon Ccljea, Siu Man Lui cljea, Vincent Ho vhjea, Hamilton Jjhjea. The Importance of Humans in Simulation: Allowing the Lure of Technology to Drive Development. Proceedings of the European Conference on Games Based Learning. 2011:343-9.
33. Flores CD, Barros P, Cazella S, Bez MR, Ieee. Leveraging the Learning Process in Health through Clinical Cases Simulator2013.

34. Islam G. Informatics Approach to Improving Surgical Skills Training: ProQuest LLC; 2013.
35. Chen Y, Wrenn J, Xu H, Spickard A, 3rd, Habermann R, Powers J, et al. Automated Assessment of Medical Students' Clinical Exposures according to AAMC Geriatric Competencies. AMIA Annual Symposium Proceedings/AMIA Symposium. 2014;2014:375-84.
36. Cao XY, Zhang P, He J, Huang GY. Building Computational Virtual Reality Environment for Anesthesia. In: Zhang C, Huang W, Shi Y, Yu PS, Zhu Y, Tian Y, et al., editors. Data Science. Lecture Notes in Computer Science. 92082015. p. 151-8.
37. Kutafina E, Laukamp D, Jonas SM. Wearable Sensors in Medical Education: Supporting Hand Hygiene Training with a Forearm EMG. Studies in Health Technology & Informatics. 2015;211:286-91.
38. Walkowski S, Lundin M, Szymas J, Lundin J. Exploring viewing behavior data from whole slide images to predict correctness of students' answers during practical exams in oral pathology. Journal of Pathology Informatics. 2015;6:28.
39. Latifi Ssluc, Gierl MJ, Boulais A-P, De Champlain AF. Using Automated Scoring to Evaluate Written Responses in English and French on a High-Stakes Clinical Competency Examination. Evaluation & the Health Professions. 2016;39(1):100-13.
40. McFadden P, Crim Aacue. Comparison of the Effectiveness of Interactive Didactic Lecture Versus Online Simulation-Based CME Programs Directed at Improving the Diagnostic Capabilities of Primary Care Practitioners. Journal of Continuing Education in the Health Professions. 2016;36(1):32-7.
41. Hamdy Hpgc, Al‐Moslih A, Tavarnesi G, Laus A. Virtual patients in problem-based learning. Medical Education. 2017;51(5):557-8.
42. Khumrin P, Ryan A, Judd T, Verspoor K. Diagnostic Machine Learning Models for Acute Abdominal Pain: Towards an e-Learning Tool for Medical Students. Studies in Health Technology & Informatics. 2017;245:447-51.
43. Alonso-Silverio GA, Perez-Escamirosa F, Bruno-Sanchez R, Ortiz-Simon JL, Munoz-Guerrero R, Minor-Martinez A, et al. Development of a Laparoscopic Box Trainer Based on Open Source Hardware and Artificial Intelligence for Objective Assessment of Surgical Psychomotor Skills. Surgical Innovation. 2018;25(4):380-8.
44. Oquendo YA, Riddle EW, Hiller D, Blinman TA, Kuchenbecker KJ. Automatically rating trainee skill at a pediatric laparoscopic suturing task. Surgical Endoscopy. 2018;32(4):1840-57.
45. Chen H, Gangaram V, Shih G. Developing a More Responsive Radiology Resident Dashboard. Journal of Digital Imaging. 2018;27:27.
46. Hayasaka Y, Fujikura T, Kashimura M. Expectations for the Next Generation of Simulated Patients Born from Thoughtful Anticipation of Artificial Intelligence-Equipped Robot. Journal of Nippon Medical School. 2018;85(6):347-9.
47. Kolachalama VB, Garg PS. Machine learning and medical education. Npj Digital Medicine. 2018;1.
48. Taherdoost H. A review of technology acceptance and adoption models and theories. Procedia Manufacturing. 2018;22:960-7.
49. Frehywot S, Vovides Y, Talib Z, Mikhail N, Ross H, Wohltjen H, et al. E-learning in medical education in resource constrained low- and middle-income countries. Human Resources for Health. 2013;11(1):4.
50. Willett TG. Current status of curriculum mapping in Canada and the UK. Medical education. 2008;42(8):786-93.
51. Hattie J, Timperley H. The power of feedback. Review of educational research. 2007;77(1):81-112.
52. Hewson MG, Little ML. Giving feedback in medical education: verification of recommended techniques. Journal of general internal medicine. 1998;13(2):111-6.
53. Mason BJ, Bruning R. Providing feedback in computer-based instruction: What the research tells us. Retrieved February. 2001;15:2007.
54. Peck C, McCall M, McLaren B, Rotem T. Continuing medical education and continuing professional development: international comparisons. BMJ: British Medical Journal. 2000;320(7232):432.
55. Shin JH, Haynes RB, Johnston ME. Effect of problem-based, self-directed undergraduate education on life-long learning. CMAJ: Canadian Medical Association Journal. 1993;148(6):969.
56. Epstein RM. Assessment in Medical Education. New England Journal of Medicine. 2007;356(4):387-96.

57. Sarrayrih MA, Ilyas M. Challenges of online exam, performances and problems for online university exam. International Journal of Computer Science Issues (IJCSI). 2013;10(1):439.
58. Ward JP, Gordon J, Field MJ, Lehmann HP. Communication and information technology in medical education. The Lancet. 2001;357(9258):792-6.
59. Van Der Vleuten CP, Schuwirth L, Driessen E, Govaerts M, Heeneman S. Twelve tips for programmatic assessment. Medical teacher. 2015;37(7):641-6.
60. Samek W, Wiegand T, Müller K-R. Explainable artificial intelligence: Understanding, visualizing and interpreting deep learning models. arXiv preprint arXiv:170808296. 2017.
61. Barrows HS, Tamblyn RM. Problem-based learning: An approach to medical education: Springer Publishing Company; 1980.
62. Government U. Data Protection Act 2018 2018 [cited 2018 10 August]. Available from: http://www.legislation.gov.uk/ukpga/2018/12/section/1.
63. Singapore PDPC. Personal Data Protection Act 2012 2012 [cited 2018 10 August]. Available from: https://www.pdpc.gov.sg/Organisations/Organisations-Overview.
64. Ellaway RH, Topps D, Pusic M. Data, Big and Small: Emerging Challenges to Medical Education Scholarship. Academic Medicine. 2019;94(1):31-6.
65. Lo B. Resolving ethical dilemmas: a guide for clinicians: Lippincott Williams & Wilkins; 2012.
66. McLean SAM. What and who are clinical ethics committees for? Journal of Medical Ethics. 2007;33(9):497-500.
67. Wartman SA, Combs CD. Reimagining Medical Education in the Age of AI. AMA Journal of Ethics. 2019;21(2):E146-52.
68. Wang JK, Roy SK, Barry M, Chang RT, Bhatt AS. Institutionalizing healthcare hackathons to promote diversity in collaboration in medicine. BMC Medical Education. 2018;18(1):269.
